# Supplementary material for: MMP-12 Inhibitors Inverse Eosinophilic Inflammation-Mediated Bronchial Fibrosis in Murine Models of Pulmonary Airway Obstruction
Source: Cells. 2025 Aug 23;14(17):1307. doi: 10.3390/cells14171307 (PMC12428497; doi:10.3390/cells14171307)
Supplement: Supplementary file 1 [file cells-14-01307-s001.zip › Suppl.figures.pdf]

Supplementary Figure-1

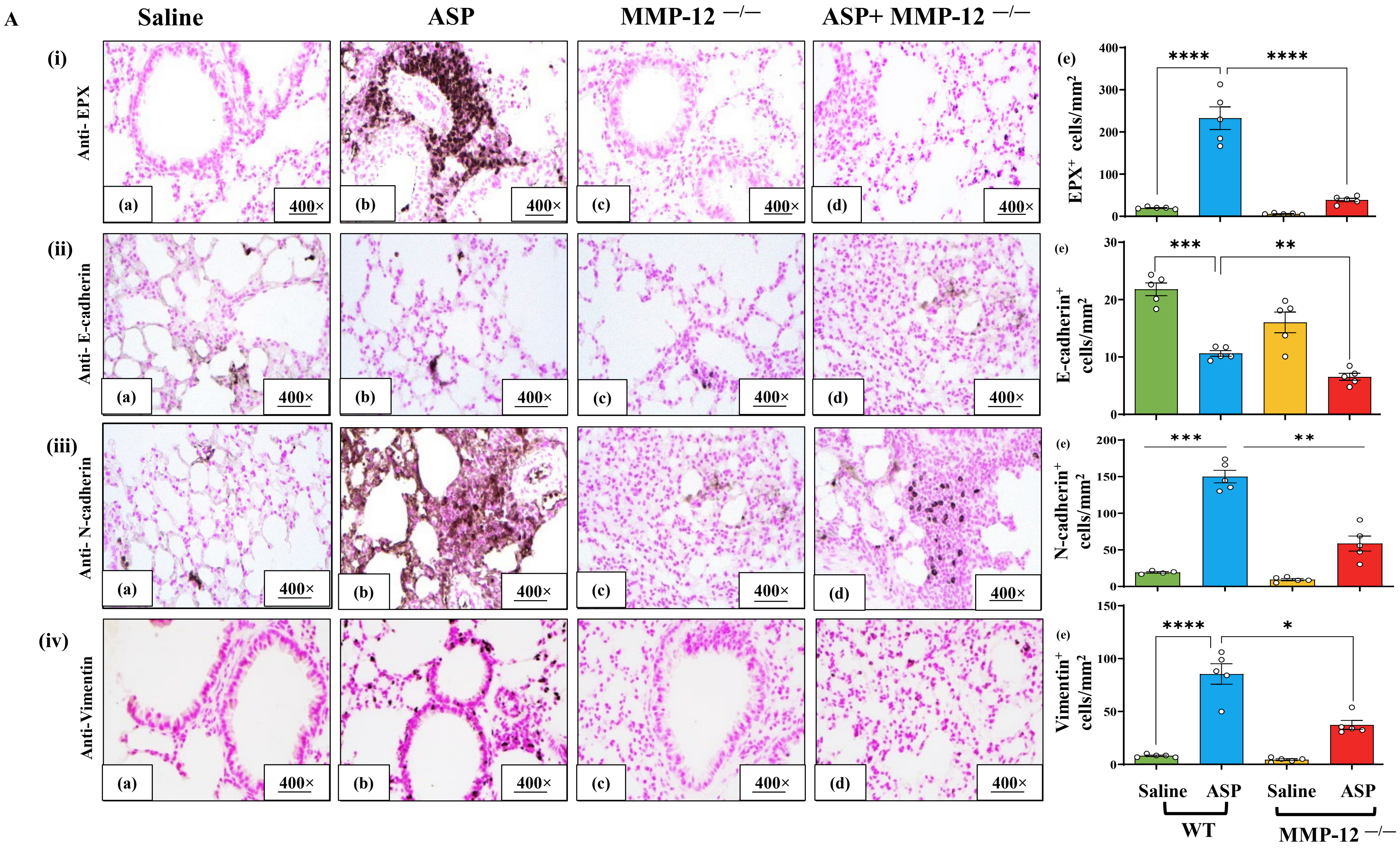

Supplementary Figure 1

MMP-12 expression correlates with eosinophilic inflammation in chronic allergen exposure

Representative lung tissue sections from saline-treated, allergen-challenged (*Af*), and MMP-12 knockout (MMP-12−/−) mice were examined to assess eosinophilic inflammation and epithelial–mesenchymal transition (EMT) markers. (i) Immunohistochemistry for eosinophil peroxidase (EPX) revealed strong eosinophilic infiltration in *Af* -exposed wild-type (WT) mice (i, b), which was markedly reduced in MMP-12−/− and *Af*-challenged MMP-12−/− groups (i, c - d) compared to saline controls (i, a). (ii) E-cadherin staining (epithelial marker) was decreased in *Af* -treated WT mice but partially preserved in MMP-12-deficient mice. (iii–iv) N-cadherin and vimentin (mesenchymal markers) were significantly increased in *Af*-challenged WT mice, while this effect was attenuated in MMP-12−/− lungs. All sections were visualized at 400× magnification. Quantification of EPX+ cells (i-e), E-cadherin+ cells (ii-e), N-cadherin+ cells (iii-e), and vimentin+ cells (iv-e) per mm^2 is shown. Data are presented as mean ± SEM (n = 4 mice/group) *$p < 0.05$, **$p < 0.01$, ***$p < 0.001$, **** $p < 0.0001$.

Supplementary Figure-2

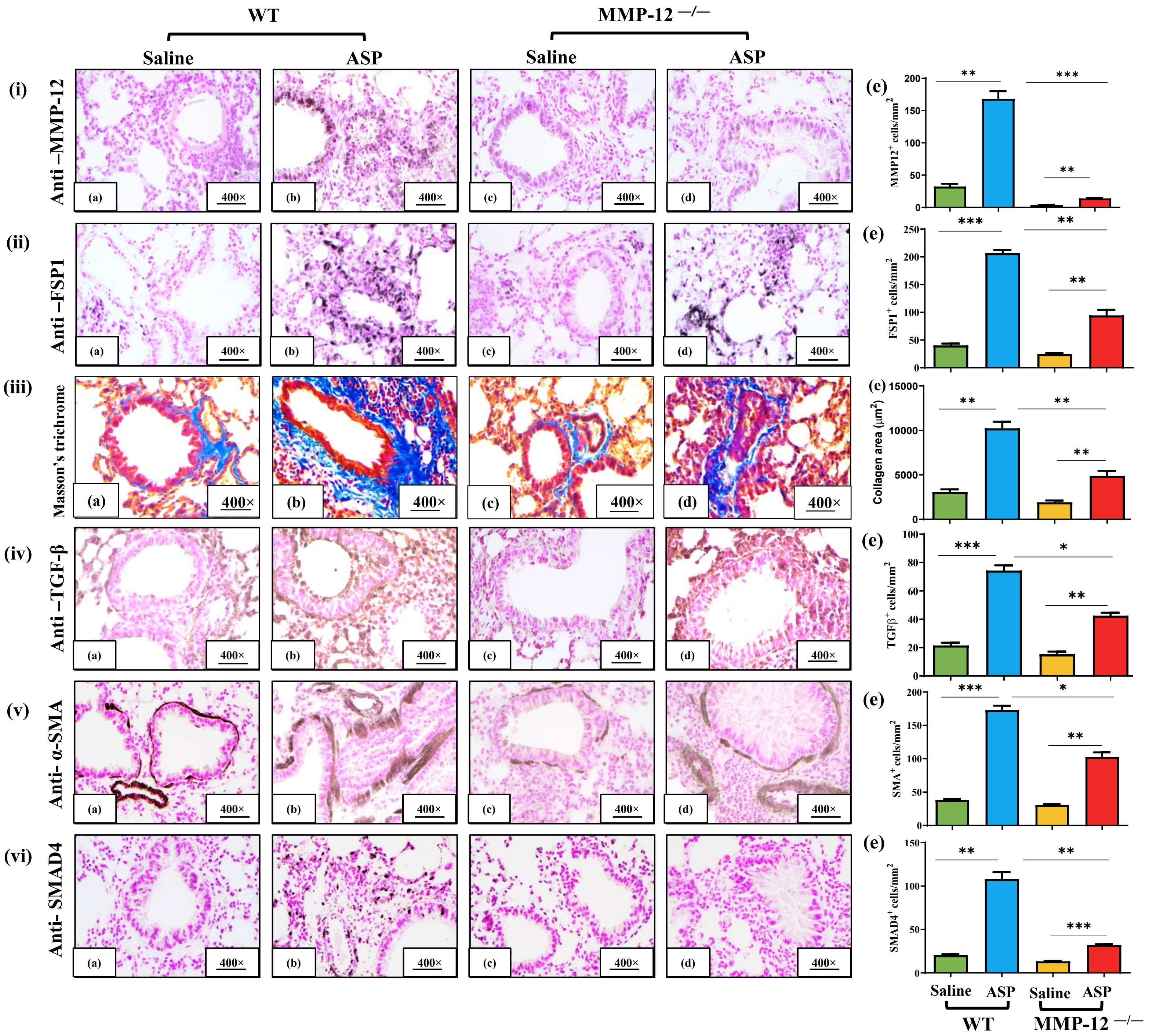

Supplementary Figure 2

MMP-12 deficiency attenuates airway remodeling and fibrosis-associated marker expression in allergen-induced lungs.

Representative lung sections from wild-type (WT) and MMP-12-deficient (MMP-12−/−) mice treated intranasally with saline or Aspergillus (*Af*) extract were stained and analyzed. (i) Immunohistochemistry (IHC) for MMP-12 shows strong expression in ASP-treated WT lungs (b) compared to saline (a), with significantly reduced expression in MMP-12−/− groups (c, d). (ii) IHC for FSP1, a fibroblast marker, demonstrates elevated expression in *Af* -treated WT lungs (b) and decreased staining in MMP-12−/− mice (d). (iii) Masson's trichrome staining highlights increased collagen deposition (blue) around airways in *Af* -exposed WT mice (b), which is markedly reduced in MMP-12−/− lungs (d). (iv) IHC for TGF-β reveals greater peribronchial staining in *Af* -exposed WT lungs (b), with reduced levels in knockout lungs (d). (v) IHC for α-SMA shows enhanced smooth muscle actin expression in *Af* -treated WT mice (b) indicative of myofibroblast activation, while knockout mice show reduced expression (d). (vi) IHC for SMAD4, a downstream mediator of TGF-β signaling, is elevated in *Af*-treated WT lungs (b) but significantly reduced in MMP-12−/− lungs (d). (e) Quantitative bar graphs corresponding to each staining represent mean ± SEM (n = 5–6 mice/group) of MMP-12+, FSP1+, collagen area (μm²), TGF-β+, α-SMA+, and SMAD4+ cells per mm². Significant reductions in profibrotic markers were observed in MMP-12−/− mice compared to WT following *Af*-exposure. Data are presented as mean ± SEM (n = 4 mice/group). $^{*}p < 0.05$, $^{**}p < 0.01$, $^{***}p < 0.001$. All images are shown at 400× magnification.

Supplementary Figure-3

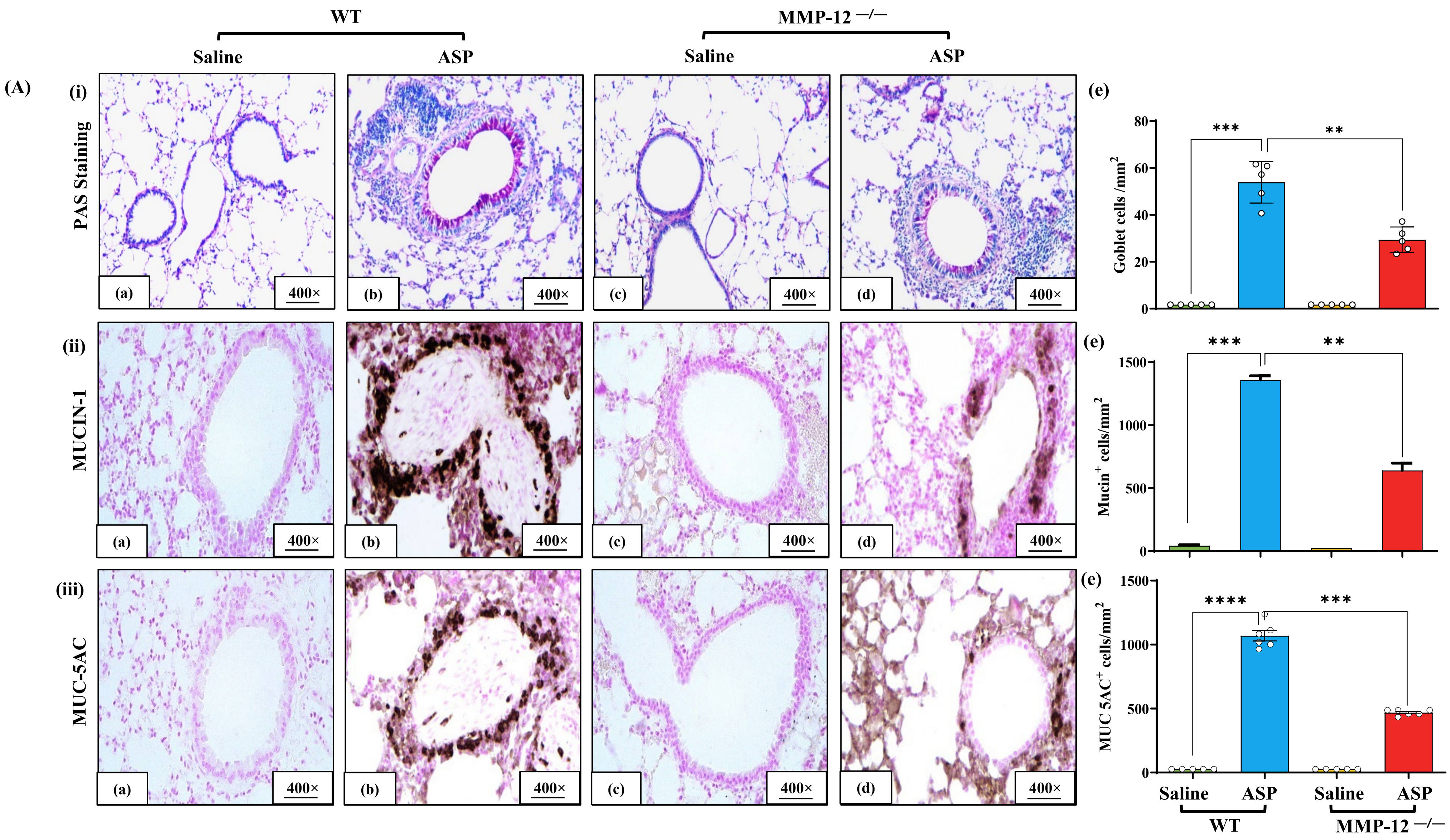

Supplementary Figure 3.

MMP-12 deficiency attenuates allergen-induced goblet cell hyperplasia and mucin production in the lungs.

Representative lung sections from wild-type (WT) and MMP-12-deficient (MMP-12−/−) mice treated with saline or allergen (*Af*) were analyzed to assess goblet cell hyperplasia and mucin expression. Periodic Acid–Schiff (PAS) staining (i) revealed a marked increase in goblet cells in *Af* -treated WT mice compared to saline controls, which was significantly attenuated in MMP-12^−/− mice. Immunohistochemistry for MUCIN-1 (ii) and MUC5AC (iii) showed robust upregulation of these mucins in *Af*-challenged WT mice, whereas MMP-12−/− mice exhibited significantly lower expression levels. All images were captured at 400× magnification. Quantitative analysis of PAS^{+} goblet cells (i-e), $MUCIN\text{-}1^{+}$ cells (ii-e), and $MUC5AC^{+}$ cells (iii-e) per mm^2 demonstrated a significant reduction in mucin-producing cells in MMP-12-deficient mice following *Af*- exposure. Data are presented as mean ± SEM (n = 4mice/group). $^{**}p < 0.01$, $^{***}p < 0.001$, $^{****}p < 0.0001$.

Supplementary Figure-4

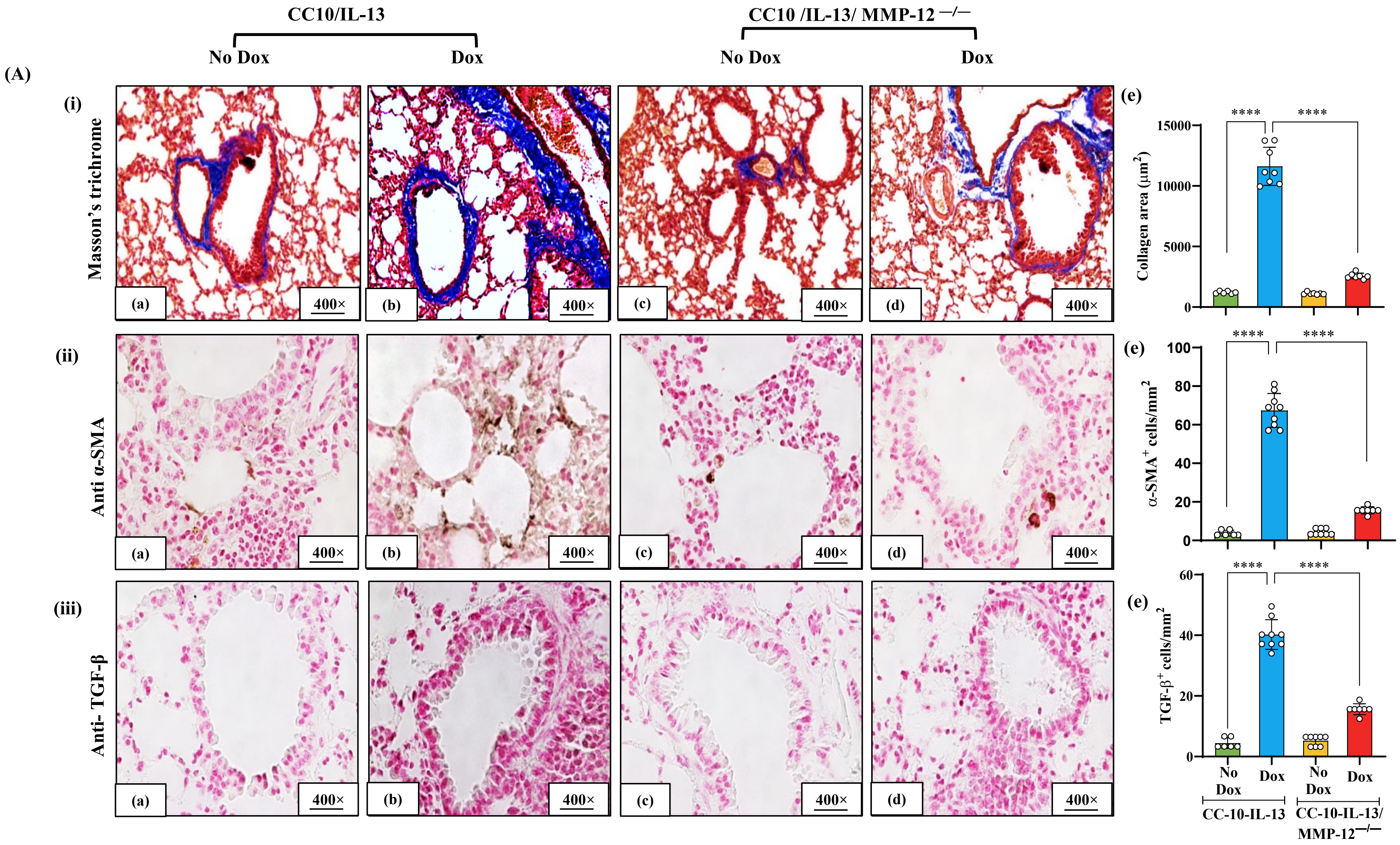

Supplementary Figure 4.

MMP-12 deficiency mitigates IL-13–induced pulmonary fibrosis and myofibroblast activation in doxycycline-inducible transgenic mice.

Lung sections from doxycycline (Dox)-inducible CC10-IL-13 and CC10-IL-13/MMP-12$^{-/-}$ bi-transgenic mice, with or without Dox administration, were analyzed for fibrosis and profibrotic markers. Masson's trichrome staining (A, i) shows extensive collagen deposition (blue) in Dox-treated CC10-IL-13 lungs (b) compared to untreated (a), while collagen staining is markedly reduced in Dox-treated CC10-IL-13/MMP-12$^{-/-}$ lungs (panel d) compared to their untreated counterparts (c). (A, ii) Immunohistochemistry (IHC) for α-SMA demonstrates increased myofibroblast activation in the Dox-induced CC10-IL-13 group (b), which is significantly attenuated in the absence of MMP-12 (d). (A, iii) IHC for TGF-β reveals upregulation in Dox-induced CC10-IL-13 lungs (b), while the Dox-treated CC10-IL-13/MMP-12$^{-/-}$ group exhibits reduced expression (d), indicating a dampened fibrotic response. Quantitative graphs (e) show significant increases in collagen area (μm^2), α-SMA$^+$, and TGF-β$^+$ cell counts in Dox-treated CC10-IL-13 lungs, with substantial reductions in the CC10-IL-13/MMP-12$^{-/-}$ mice upon Dox exposure. Data are presented as mean ± SEM (n = 4 mice/group), ****$p < 0.0001$. All images captured at 400× magnification.

Supplementary Figure-5

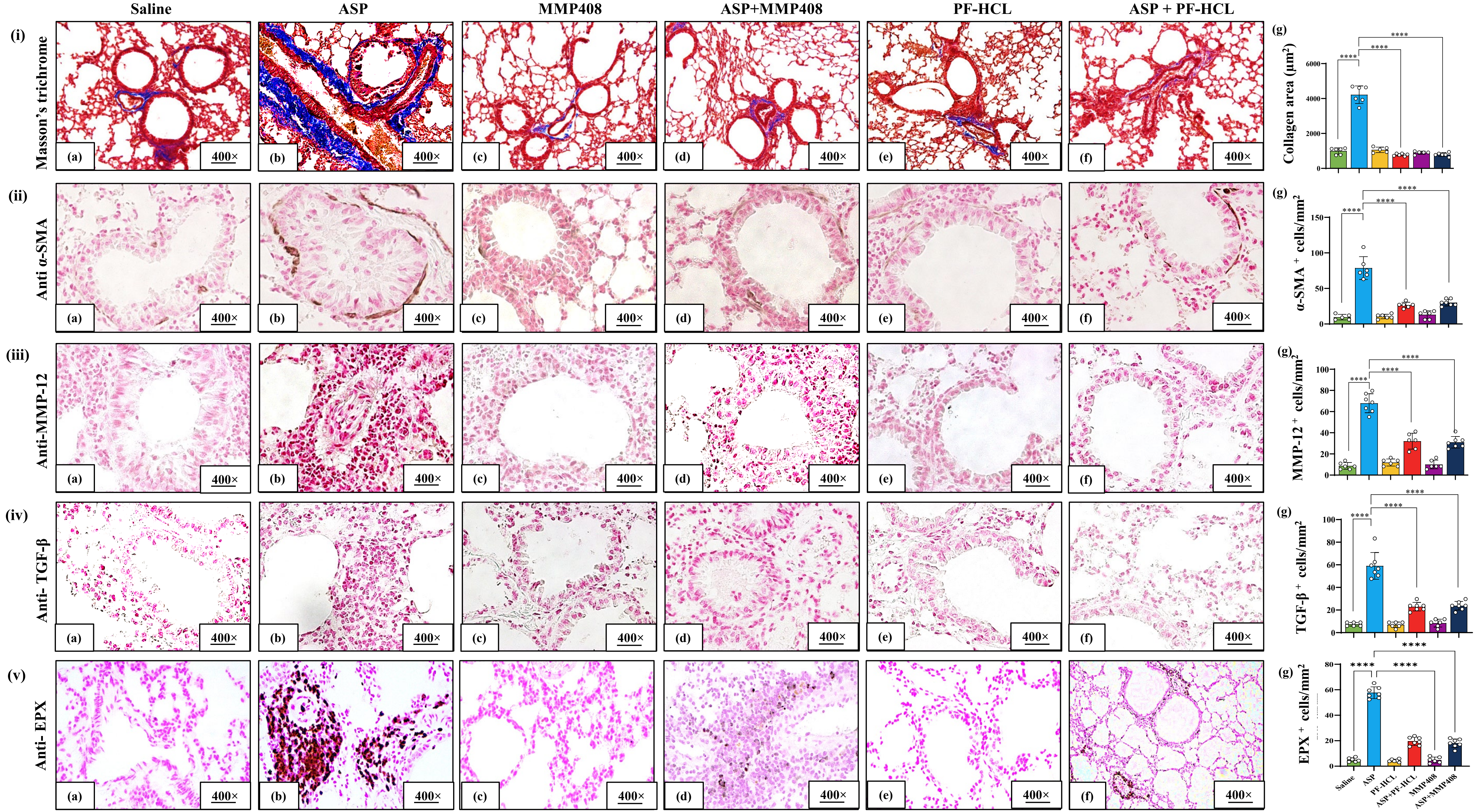

Supplementary Figure 5.

Pharmacological inhibition of MMP-12 attenuates allergen-induced lung fibrosis and expression of profibrotic markers.

Lung sections from mice treated with saline, *Aspergillus* (ASP) extract, MMP-12 inhibitors (MMP408 or PF-HCL), or a combination of ASP with MMP-12 inhibitors were analyzed to assess the effect of MMP-12 inhibition on allergen-induced pulmonary fibrosis and profibrotic responses. Masson's trichrome staining (i) shows marked collagen accumulation in ASP-treated lungs (b), which is significantly reduced following treatment with either MMP408 (d) or PF-HCL (f). Immunohistochemistry for α-SMA (ii) reveals increased myofibroblast activation in ASP-treated mice, with reduced α-SMA expression in the inhibitor-treated groups. MMP-12 staining (iii) confirms strong induction by ASP, which is suppressed by MMP408 and PF-HCL. Similarly, TGF-β staining (iv) is elevated in ASP-treated lungs and reduced upon MMP-12 inhibitor treatment. Quantitative analysis (i-iv, g) demonstrates that collagen area, and numbers of α-SMA^{+}, MMP-12^{+}, and TGF-β^{+} cells are significantly increased in ASP-exposed lungs and significantly attenuated with either MMP408 or PF-HCL treatment. Eosinophils were also showed by EPX attaining (v), followed by quantification. Data represent mean ± SEM (n = 4 mice/group), ****$p <$ 0.0001. All images were acquired at 400× magnification.

Supplementary Figure-6

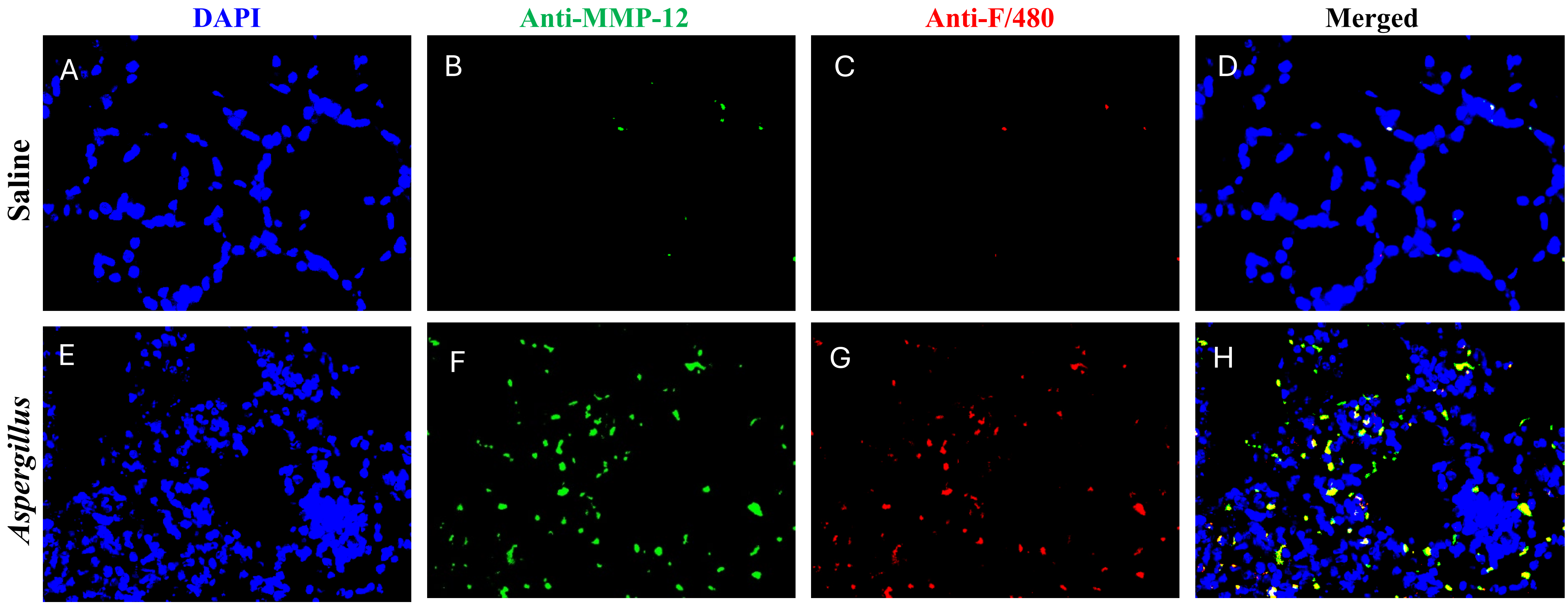

The immunofluorescence analysis was performed using DAPI, anti-MMP-12, anti-F4/80 on the lung tissue sections of saline and *Af*-challenged mice. DAPI mounted merged photomicrograph for MMP-12 expression on lung fibroblast in saline (A-D) and *Af-challenged* (E-H) mice are shown. Images were captured and presented in 400x of original magnification.

Supplementary Figure-7 original blots

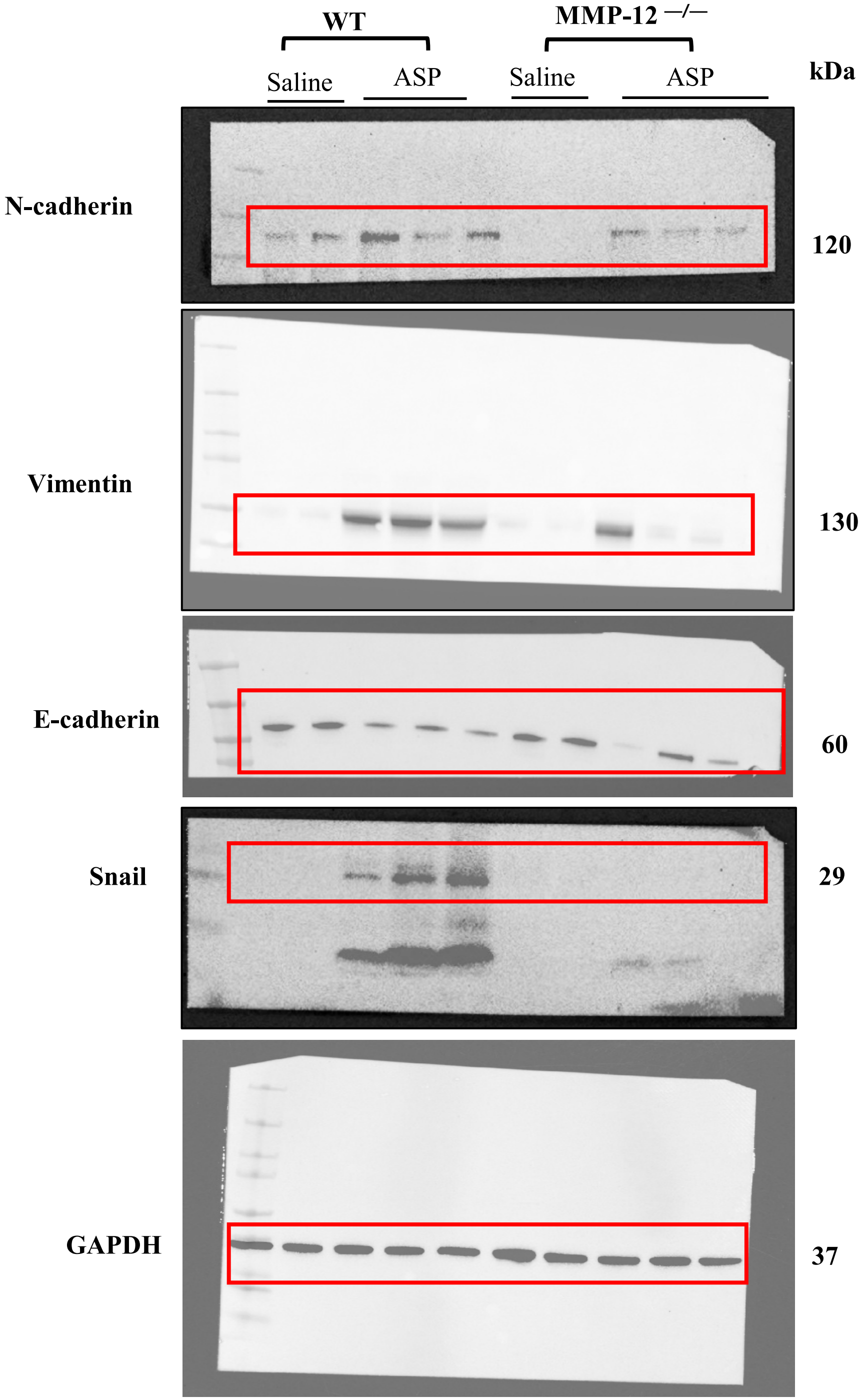

Supplementary Figure-8 original blots

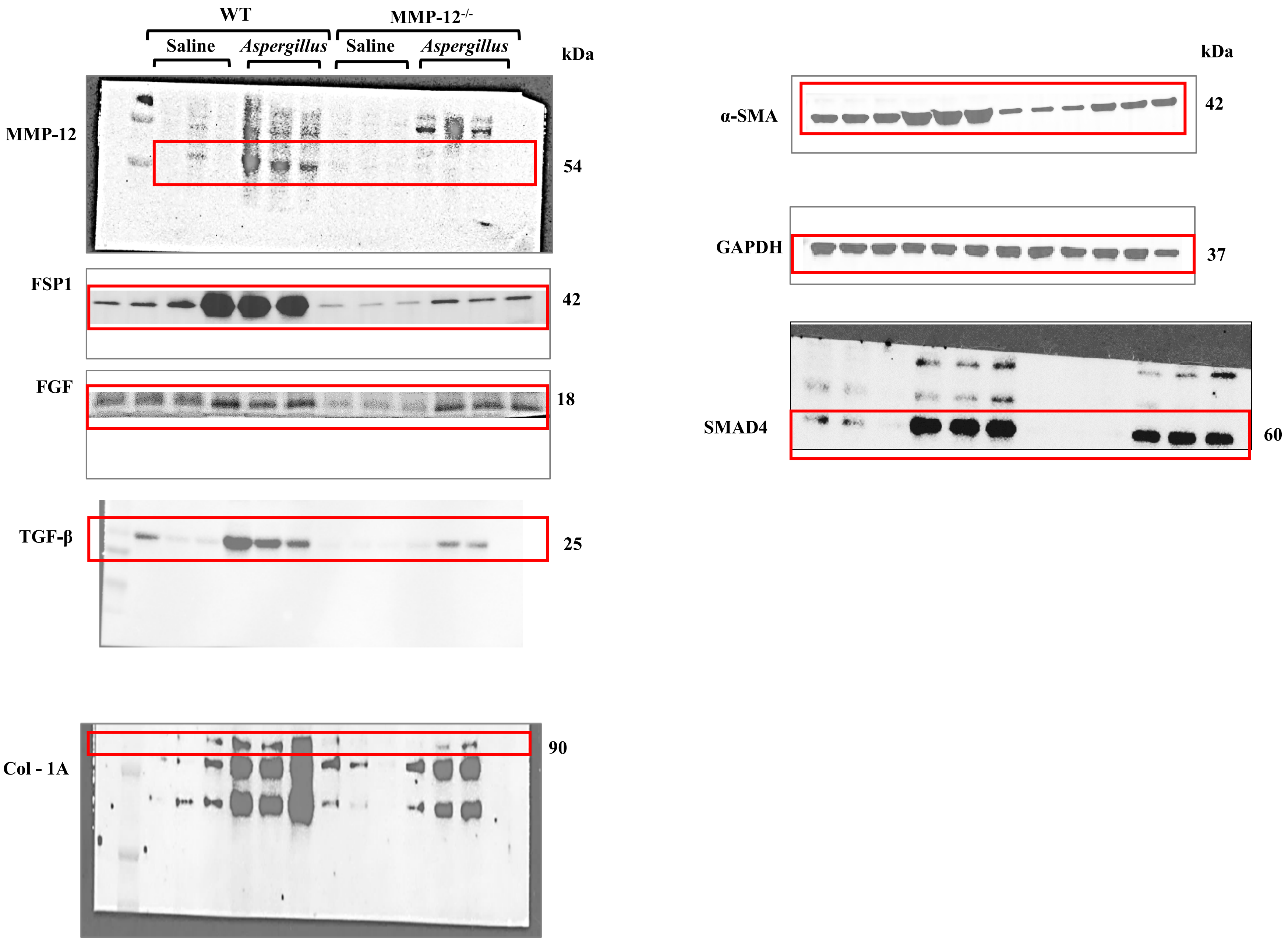

Supplementary Figure-9 original blots

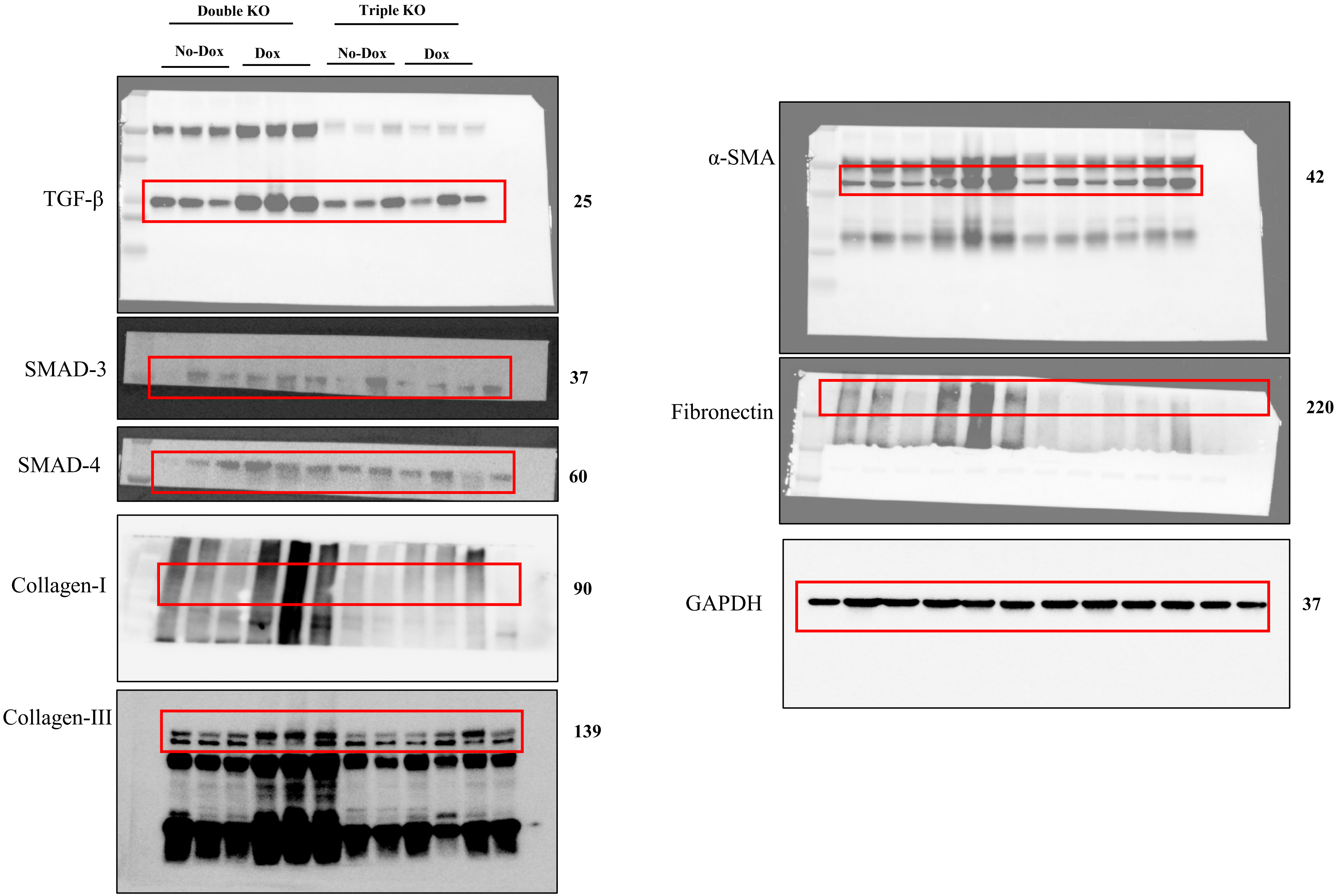

Supplementary Figure-10 original blots

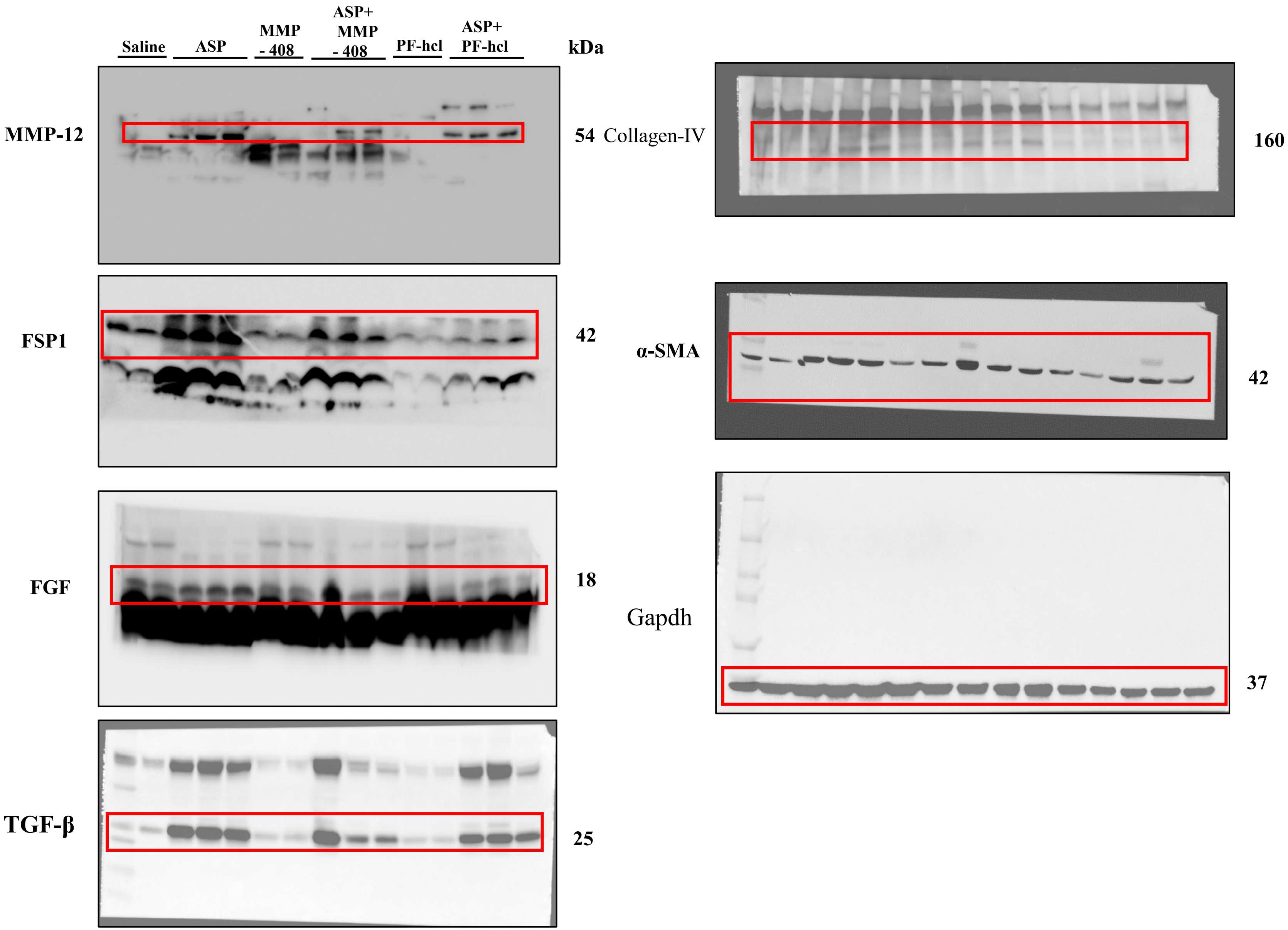

Supplementary table-1- List of antibodies, dilutions used in western blotting and immunohistochemistry

S.No.	Antibody	Catalog number	Supplier	Dilutions	
				WB	IHC
1	MMP-12	22989-1-AP	Proteintech Group, Inc	1:1000	1:200
2	Col1A	sc-59772	Santa Cruz Biotechnology, Inc	1:1000	NA
3	GAPDH	60004-1-Ig	Proteintech Group, Inc	1:1000	NA
4	TGF-β	26155-1-AP	Proteintech Group, Inc	1:1000	1:200
5	SMAD4	46535	CST	1:1000	1:200
6	Collagen III	22734-1-AP	Proteintech Group, Inc	1:1000	NA
7	Fibronectin	sc-8422	Santa Cruz Biotechnology, Inc	1:2000	NA
8	Collagen IV	sc-59814	Santa Cruz Biotechnology, Inc	1:1000	NA
9	SMAD3	9523	CST	1:1000	1:200
10	Snail	3879	CST	1:1000	NA
11	FGF	11234-1-AP	Proteintech Group, Inc	1:1000	1:200
12	FSP1	20886-1-AP	Proteintech Group, Inc	1:1000	1:200
13	E-Cadherin	sc-21791	Santa Cruz Biotechnology, Inc	1:1000	1:200
14	N-Cadherin	sc-59987		1:1000	1:200
15	Vimentin	sc-6260		1:1000	1:200
16	α-SMA	14395-1-AP	Proteintech Group, Inc	1:1000	1:200
17	EPX	Clone (MM25.82.2.1)	Mayo Clinic	NA	1:5000
18	Anti-rabbit IgG, HRP	7074	Santa Cruz Biotechnology, Inc	1:5000	NA
19	Anti-mouse IgG, HRP	7076	Santa Cruz Biotechnology, Inc	1:5000	NA
20	Goat Anti-Mouse IgG	BA-9200	Vector Laboratories, Inc.	NA	1:250
21	Goat Anti-Rat IgG	BA-9400	Vector Laboratories, Inc.	NA	1:250
22	Goat Anti-Rabbit IgG	BA-1000	Vector Laboratories, Inc.	NA	1:250

Supplementary Table 2 statistical tests applied

Figure 1B

Unpaired t test	
P value	<0.0001
P value summary	****

Figure 1C

Unpaired t test	
P value	0.0023
P value summary	**

Figure 1D

Unpaired t test	
P value	0.0001
P value summary	***

Figure 1E

Unpaired t test	
P value	0.0019
P value summary	**

Figure 1F

Dunnett's multiple comparisons test	Summary	Adjusted P Value
WT ASP vs. WT Saline	****	<0.0001
WT ASP vs. MMP12$^{-/-}$ Saline	****	<0.0001
WT ASP vs. MMP12$^{-/-}$ ASP	****	<0.0001

Figure 1G

Dunnett's multiple comparisons test	Summary	Adjusted P Value
WT ASP vs. Saline	****	<0.0001
WT ASP vs. MMP12$^{-/-}$Saline	****	<0.0001
WT ASP vs. MMP12$^{-/-}$ASP	****	<0.0001

Figure 1H

Tukey's multiple comparisons test	Summary	Adjusted P Value
WT Saline vs. WT *A.fumigatus*	****	<0.0001
WT Saline vs. MMP12$^{-/-}$Saline	****	<0.0001
WT Saline vs. MMP12$^{-/-}$*A.fumigatus*	****	<0.0001
WT *A.fumigatus* vs. MMP12$^{-/-}$Saline	****	<0.0001
WT *A.fumigatus* vs. MMP12$^{-/-}$*A.fumigatus*	****	<0.0001
MMP12$^{-/-}$Saline vs. MMP12$^{-/-}$*A.fumigatus*	****	<0.0001

Supplementary Table 3 statistical tests applied

Figure 2A

Unpaired t test	
P value	<0.0001
P value summary	****

Figure 2B

Unpaired t test	
P value	<0.0001
P value summary	****

Figure 2C

Unpaired t test	
P value	0.0005
P value summary	***

Figure 2D

Unpaired t test	
P value	<0.0001
P value summary	****

Figure 2E

Unpaired t test	
P value	<0.0001
P value summary	****

Figure 2F

Unpaired t test	
P value	<0.0001
P value summary	****

Figure 2G

Tukey's multiple comparisons test	Summary	Adjusted P Value
WT Saline vs. WT ASP	****	<0.0001
WT ASP vs. MMP12$^{-/-}$ ASP	****	<0.0001

Figure 2H

Dunnett's multiple comparisons test	Summary	Adjusted P Value
WT Saline vs. WT ASP	****	<0.0001
WT ASP vs. MMP12$^{-/-}$ ASP	****	<0.0001

Supplementary Table 4 statistical tests applied

Figure 3A

Unpaired t test	
P value	0.0008
P value summary	***

Figure 3B

Unpaired t test	
P value	<0.0001
P value summary	****

Figure 3C

Unpaired t test	
P value	<0.0001
P value summary	****

Figure 3D

One-way ANOVA		Ordinary
Dunnett's multiple comparisons test	Summary	Adjusted P Value
A.fumigatus vs. Saline	****	<0.0001
A.fumigatus vs. MMP12 KO Saline	****	<0.0001
A.fumigatus vs. MMP12 KO *A.fumigatus*	****	<0.0001

Figure 3E

One-way ANOVA		Ordinary
Dunnett's multiple comparisons test	Summary	Adjusted P Value
A.fumigatus vs. Saline	****	<0.0001
A.fumigatus vs. MMP12 KO Saline	****	<0.0001
A.fumigatus vs. MMP12 KO *A.fumigatus*	****	<0.0001

Figure 3F

One-way ANOVA summary		
Tukey's multiple comparisons test	Summary	Adjusted P Value
WT Saline vs. WT ASP	****	<0.0001
WT Saline vs. MMP12$^{-/-}$ Saline	****	<0.0001
WT Saline vs. MMP12$^{-/-}$ ASP	ns	0.1121
WT ASP vs. MMP12$^{-/-}$ Saline	****	<0.0001
WT ASP vs. MMP12$^{-/-}$ ASP	****	<0.0001
MMP12$^{-/-}$ Saline vs. MMP12$^{-/-}$ ASP	****	<0.0001

Supplementary Table 5 statistical tests applied

Figure 4B

Unpaired t test	
P value	<0.0001
P value summary	****

Figure 4C

Unpaired t test	
P value	<0.0001
P value summary	****

Figure 4D

Unpaired t test	
P value	<0.0001
P value summary	****

Figure 4E

Dunnett's multiple comparisons test	Summary	Adjusted P Value
CC10IL13 Dox vs. CC10IL13 No Dox	****	<0.0001
CC10IL13 Dox vs. CC10IL13-MMP12$^{-/-}$ No Dox	****	<0.0001
CC10IL13 Dox vs. CC10IL13-MMP12$^{-/-}$ Dox	****	<0.0001

Figure 4F

Tukey's multiple comparisons test	Summary	Adjusted P Value
CC10/IL-13$^{-/-}$ No Dox vs. CC10/IL-13$^{-/-}$Dox	****	<0.0001
CC10/IL-13$^{-/-}$ No Dox vs. CC10/IL-13$^{-/-}$/MMP-12 $^{-/-}$NoDox	ns	0.2617
CC10/IL-13$^{-/-}$ No Dox vs. CC10/IL-13$^{-/-}$/MMP-12 $^{-/-}$ Dox	ns	0.6681
CC10/IL-13$^{-/-}$Dox vs. CC10/IL-13$^{-/-}$/MMP-12 $^{-/-}$NoDox	****	<0.0001
CC10/IL-13$^{-/-}$Dox vs. CC10/IL-13$^{-/-}$/MMP-12 $^{-/-}$ Dox	****	<0.0001
CC10/IL-13$^{-/-}$/MMP-12 $^{-/-}$NoDox vs. CC10/IL-13$^{-/-}$/MMP-12 $^{-/-}$ Dox	*	0.0428

Supplementary Table 6 statistical tests applied

Figure 5B

Tukey's multiple comparisons test	Summary	Adjusted P Value
ASP vs. Saline	****	<0.0001
ASP vs. ASP+PF-HCL	****	<0.0001
ASP vs. ASP+MMP408	****	<0.0001

Figure 5C

Tukey's multiple comparisons test	Summary	Adjusted P Value
ASP vs. Saline	****	<0.0001
ASP vs. ASP+PF-HCL	****	<0.0001
ASP vs. ASP+MMP408	****	<0.0001

Figure 5D

Tukey's multiple comparisons test	Summary	Adjusted P Value
ASP vs. Saline	****	<0.0001
ASP vs. ASP+PF-HCL	****	<0.0001
ASP vs. ASP+MMP408	****	<0.0001

Figure 5E

Tukey's multiple comparisons test	Summary	Adjusted P Value
ASP vs. Saline	****	<0.0001
ASP vs. ASP+PF-HCL	****	<0.0001
ASP vs. ASP+MMP408	****	<0.0001

Figure 5F

Dunnett's multiple comparisons test	Summary	Adjusted P Value
A.fumigatus vs. Saline	****	<0.0001
A.fumigatus vs. MMP408	****	<0.0001
A.fumigatus vs. MMP408+*A.fumigatus*	****	<0.0001
A.fumigatus vs. PF-00356231	****	<0.0001
A.fumigatus vs. PF-00356231+*A.fumigatus*	****	<0.0001

Supplementary Table 7 statistical tests applied

Figure 6B

Dunnett's multiple comparisons test	Summary	Adjusted P Value
ASP vs. ASP+MMP408	****	<0.0001
ASP vs. ASP+PF-HCL	****	<0.0001

Figure 6C

Dunnett's multiple comparisons test	Summary	Adjusted P Value
ASP vs. Saline	***	0.0005
ASP vs. PF-HCL	***	0.0005
ASP vs. ASP+PF-HCL	*	0.0386
ASP vs. MMP408	***	0.0004
ASP vs. ASP+MMP408	**	0.0065

Figure 6D

Dunnett's multiple comparisons test	Summary	Adjusted P Value
A.fumigatus vs. Saline	****	<0.0001
A.fumigatus vs. MMP12 KO Saline	****	<0.0001
A.fumigatus vs. MMP12 KO *A.fumigatus*	****	<0.0001

Figure 6E

Dunnett's multiple comparisons test	Summary	Adjusted P Value
A.fumigatus vs. CON	****	<0.0001
A.fumigatus vs. PF-00356231	****	<0.0001
A.fumigatus vs. PF-00356231+*A.fumigatus*	****	<0.0001
A.fumigatus vs. MMP408	****	<0.0001
A.fumigatus vs. MMP408+*A.fumigatus*	***	0.0002

Supplementary Table 8 Statistical Test Applied in all figures

Figure	Measurement Type	Statistical Test Used
**Figure 1B**	IHC (EPX+ cells)	Unpaired t test
**Figure 1C–E**	IHC (EMT markers)	Unpaired t test
**Figure 1F**	Western blot (EMT proteins)	One-way ANOVA; Dunn’s multiple comparisons test
**Figure 1G–H**	ELISA (cytokines)	One-way ANOVA; Dunn’s/Tukey’s multiple comparisons test
**Figure 2A–F**	IHC (fibrosis markers)	Unpaired t test
**Figure 2G**	Western blot (fibrosis markers)	One-way ANOVA; Tukey’s multiple comparisons test
**Figure 2H**	ELISA (TGF-β)	One-way ANOVA; Dunn’s multiple comparisons test
**Figure 3A–C**	IHC (mucus/goblet cell markers)	Unpaired t test
**Figure 3D–E**	Lung function (RI, Cdyn)	One-way ANOVA; Dunn’s multiple comparisons test
**Figure 3F**	ELISA (IL-13)	One-way ANOVA; Tukey’s multiple comparisons test
**Figure 4B–D**	IHC (fibrosis markers)	Unpaired t test
**Figure 4E**	Western blot (fibrosis markers)	One-way ANOVA; Dunn’s multiple comparisons test
**Figure 4F**	ELISA (IL-13)	One-way ANOVA; Tukey’s multiple comparisons test
**Figure 5B–E**	IHC (fibrosis markers)	One-way ANOVA; Tukey’s multiple comparisons test
**Figure 5F**	Western blot (fibrosis markers)	One-way ANOVA; Dunn’s multiple comparisons test
**Figure 6A–B**	IHC (goblet cells)	One-way ANOVA; Dunn’s multiple comparisons test
**Figure 6C–D**	Lung function (RI, Cdyn)	One-way ANOVA; Dunn’s multiple comparisons test
**Figure 6E**	ELISA (IL-13)	One-way ANOVA; Dunn’s multiple comparisons test

Supplementary Table 9 normality test results

Figure 1B

Test for normal distribution	WT ASP	MMP12$^{-/-}$ ASP
Shapiro-Wilk test		
W	0.9564	0.9659
P value	0.7829	0.8483
Passed normality test (alpha=0.05)?	Yes	Yes

Figure 1C

Test for normal distribution	WT ASP	MMP12$^{-/-}$ ASP
Shapiro-Wilk test		
W	0.8999	0.9681
P value	0.4096	0.8630
Passed normality test (alpha=0.05)?	Yes	Yes

Figure 1D

Test for normal distribution	WT ASP	MMP12$^{-/-}$ ASP
Shapiro-Wilk test		
W	0.9079	0.9926
P value	0.4551	0.9879
Passed normality test (alpha=0.05)?	Yes	Yes

Figure 1E

Test for normal distribution	WT ASP	MMP12$^{-/-}$ ASP
Shapiro-Wilk test		
W	0.8909	0.7594
P value	0.3617	0.1290
Passed normality test (alpha=0.05)?	Yes	Yes

Figure 1F-ii

Test for normal distribution	WT Saline	WT ASP	MMP12$^{-/-}$ Saline	MMP12$^{-/-}$ ASP
Shapiro-Wilk test				
W	0.8378	0.8871	0.8506	0.9057
P value	0.1892	0.3699	0.2282	0.4601
Passed normality test (alpha=0.05)?	Yes	Yes	Yes	Yes

Figure 1G

Test for normal distribution	WT Saline	WT ASP	MMP12$^{-/-}$ Saline	MMP12$^{-/-}$ ASP
Shapiro-Wilk test				
W	0.9700	0.8845	0.9734	0.9498
P value	0.6673	0.3377	0.6871	0.5683
Passed normality test (alpha=0.05)	Yes	Yes	Yes	Yes

Figure 1H

Test for normal distribution	WT Saline	WT ASP	MMP12$^{-/-}$ Saline	MMP12$^{-/-}$ ASP
Shapiro-Wilk test				
W	0.9336	0.9343	0.9277	0.9437
P value	0.5021	0.5051	0.4801	0.5423
Passed normality test (alpha=0.05)?	Yes	Yes	Yes	Yes

Supplementary Table 10 normality test results

Figure 2A

Test for normal distribution	WT ASP	MMP12$^{-/-}$ ASP
Shapiro-Wilk test		
W	0.8035	0.9186
P value	0.0866	0.5210
Passed normality test (alpha=0.05)?	Yes	Yes

Figure 2B

Test for normal distribution	WT ASP	MMP12$^{-/-}$ ASP
Shapiro-Wilk test		
W	0.9488	0.9003
P value	0.7287	0.4113
Passed normality test (alpha=0.05)?	Yes	Yes

Figure 2C

Test for normal distribution	WT ASP	MMP12$^{-/-}$ ASP
Shapiro-Wilk test		
W	0.9540	0.9150
P value	0.7655	0.4985
Passed normality test (alpha=0.05)?	Yes	Yes

Figure 2D

Test for normal distribution	WT ASP	MMP12$^{-/-}$ ASP
Shapiro-Wilk test		
W	0.8771	0.9578
P value	0.2964	0.7925
Passed normality test (alpha=0.05)?	Yes	Yes

Figure 2E

Test for normal distribution	WT ASP	MMP12$^{-/-}$ ASP
Shapiro-Wilk test		
W	0.8939	0.8965
P value	0.3771	0.3910
Passed normality test (alpha=0.05)?	Yes	Yes

Figure 2F

Test for normal distribution	WT ASP	MMP12$^{-/-}$ ASP
Shapiro-Wilk test		
W	0.8863	0.8631
P value	0.3389	0.2395
Passed normality test (alpha=0.05)?	Yes	Yes

Figure 2G-ii

Test for normal distribution	WT Saline	WT ASP	MMP12$^{-/-}$ Saline	MMP12$^{-/-}$ ASP
Shapiro-Wilk test				
W	Invalid input data	0.8602	0.7530	0.9724
P value		0.1520	0.0593	0.9151
Passed normality test (alpha=0.05)		Yes	Yes	Yes

Figure 2H

Test for normal distribution	WT Saline	WT ASP	MMP12$^{-/-}$ Saline	MMP12$^{-/-}$ ASP
Shapiro-Wilk test				
W	0.8611	0.7263	0.9232	0.9438
P value	0.2324	0.0602	0.5510	0.6926
Passed normality test (alpha=0.05)	Yes	Yes	Yes	Yes

Supplementary Table 11 normality test results

Figure 3A

Test for normal distribution	WT ASP	$MMP12^{-/-}$ ASP
Shapiro-Wilk test		
W	0.8864	0.9683
P value	0.3395	0.8645
Passed normality test (alpha=0.05)?	Yes	Yes

Figure 3B

Test for normal distribution	WT ASP	$MMP12^{-/-}$ ASP
Shapiro-Wilk test		
W	0.9416	0.9294
P value	0.6722	0.5757
Passed normality test (alpha=0.05)?	Yes	Yes

Figure 3C

Test for normal distribution		
Shapiro-Wilk test		
W	0.9326	0.8385
P value	0.6005	0.1267
Passed normality test (alpha=0.05)?	Yes	Yes

Figure 3D

Test for normal distribution	WT Saline	WT ASP	$MMP12^{-/-}$ Saline	$MMP12^{-/-}$ ASP
Shapiro-Wilk test				
W	0.9373	0.9535	0.8338	0.9087
P value	0.6469	0.7625	0.1485	0.4599
Passed normality test (alpha=0.05)?	Yes	Yes	Yes	Yes

Figure 3E

Test for normal distribution	WT Saline	WT ASP	$MMP12^{-/-}$ Saline	$MMP12^{-/-}$ ASP
Shapiro-Wilk test				
W	0.9576	0.6251	0.8646	0.8778
P value	0.7911	0.0513	0.2453	0.2995
Passed normality test (alpha=0.05)?	Yes	Yes	Yes	Yes

Figure 3F

Test for normal distribution	WT Saline	WT ASP	$MMP12^{-/-}$ Saline	$MMP12^{-/-}$ ASP
Shapiro-Wilk test				
W	0.9410	0.8064	0.9125	0.8531
P value	0.6672	0.0671	0.4529	0.1666
Passed normality test (alpha=0.05)?	Yes	Yes	Yes	Yes

Supplementary Table 12 normality test results

Figure 4B

Test for normal distribution	CC-10/IL-13 Dox	CC-10-IL-13/ MMP-12 $^{-/-}$ Dox
Shapiro-Wilk test		
W	0.8550	0.9167
P value	0.1071	0.4439
Passed normality test (alpha=0.05)?	Yes	Yes

Figure 4C

Test for normal distribution	CC-10/IL-13 Dox	CC-10-IL-13/ MMP-12 $^{-/-}$ Dox
Shapiro-Wilk test		
W	0.9293	0.8065
P value	0.4743	0.0538
Passed normality test (alpha=0.05)?	Yes	Yes

Figure 4D

Test for normal distribution	CC-10/IL-13 Dox	CC-10-IL-13/ MMP-12 $^{-/-}$ Dox
Shapiro-Wilk test		
W	0.8863	0.7965
P value	0.1828	0.0511
Passed normality test (alpha=0.05)?	Yes	Yes

Figure 4E-ii

	**CC-10/IL-13**		**CC-10-IL-13/ MMP-12 $^{-/-}$**	
Test for normal distribution	No Dox	Dox	No Dox	Dox
Shapiro-Wilk test				
W	0.6530	0.9135	Invalid input data	0.8650
P value	0.053	0.4207		0.1676
Passed normality test (alpha=0.05)	Yes	Yes		Yes

Figure 4F

	**CC-10/IL-13**		**CC-10-IL-13/ MMP-12 $^{-/-}$**	
Test for normal distribution	No Dox	Dox	No Dox	Dox
Shapiro-Wilk test				
W	0.8484	0.9127	0.9671	0.9237
P value	0.1528	0.4842	0.8563	0.5538
Passed normality test (alpha=0.05)?	Yes	Yes	Yes	Yes

Supplementary Table 13 normality test results

Figure 5B

Test for normal distribution	**Saline**	ASP	PF-HCL	ASP+PF-HCL	MMP408	ASP+MMP408
Shapiro-Wilk test						
W	0.9076	0.9714	0.9067	0.9338	0.8663	0.9138
P value	0.4207	0.9083	0.4150	0.6097	0.2117	0.4227
Passed normality test (alpha=0.05)	Yes	Yes	Yes	Yes	Yes	Yes

Figure 5C

Test for normal distribution	**Saline**	ASP	PF-HCL	ASP+PF-HCL	MMP408	ASP+MMP408
Shapiro-Wilk test						
W	0.7904	0.8628	0.9527	0.9290	0.8205	0.9396
P value	0.0752	0.1988	0.7568	0.5724	0.1178	0.6563
Passed normality test (alpha=0.05)	Yes	Yes	Yes	Yes	Yes	Yes

Figure 5E

Test for normal distribution	**Saline**	ASP	PF-HCL	ASP+PF-HCL	MMP408	ASP+MMP408
Shapiro-Wilk test						
W	0.9020	0.8683	0.9600	0.9212	0.7676	0.8964
P value	0.4211	0.2194	0.8201	0.5141	0.0593	0.3100
Passed normality test (alpha=0.05)	Yes	Yes	Yes	Yes	Yes	Yes

Figure 5D

Test for normal distribution	**Saline**	ASP	PF-HCL	ASP+PF-HCL	MMP408	ASP+MMP408
Shapiro-Wilk test						
W	0.7827	0.8485	0.8216	0.9146	0.9076	0.9601
P value	0.0740	0.1192	0.0911	0.4287	0.4207	0.8192
Passed normality test (alpha=0.05)?	Yes	Yes	Yes	Yes	Yes	Yes

Figure 5F-ii

Test for normal distribution	**Saline**	ASP	PF-HCL	ASP+PF-HCL	MMP408	ASP+MMP408
Shapiro-Wilk test						
W		0.9788		0.9500		0.7943
P value		0.9454		0.7403		0.0522
Passed normality test (alpha=0.05)?		Yes		Yes		Yes

Supplementary Table 14 normality test results

Figure 6B

Test for normal distribution	ASP	ASP+PF-HCL	ASP+MMP408
Shapiro-Wilk test			
W	0.9619	0.9081	0.9371
P value	0.8339	0.4238	0.6361
Passed normality test (alpha=0.05)?	Yes	Yes	Yes

Figure 6C

Test for normal distribution	**Saline**	ASP	PF-HCL	ASP+PF-HCL	MMP408	ASP+MMP408
Shapiro-Wilk test						
W	0.9258	0.8235	0.9354	0.9628	0.9245	0.9371
P value	0.5678	0.1243	0.6336	0.8276	0.5595	0.6455
Passed normality test (alpha=0.05)?	Yes	Yes	Yes	Yes	Yes	Yes

Figure 6D

Test for normal distribution	**Saline**	ASP	PF-HCL	ASP+PF-HCL	MMP408	ASP+MMP408
Shapiro-Wilk test						
W	0.9259	0.8090	0.8412	0.7816	0.8349	0.7997
P value	0.5690	0.0830	0.1683	0.0730	0.1513	0.0806
Passed normality test (alpha=0.05)?	Yes	Yes	Yes	Yes	Yes	Yes
P value summary	ns	ns	ns	ns	ns	ns

Figure 6E

Test for normal distribution	**Saline**	ASP	PF-HCL	ASP+PF-HCL	MMP408	ASP+MMP408
Shapiro-Wilk test						
W	0.9214	0.9584	0.8805	0.9368	0.9386	0.9487
P value	0.5154	0.8072	0.2715	0.6334	0.6476	0.7295
Passed normality test (alpha=0.05)?	Yes	Yes	Yes	Yes	Yes	Yes
P value summary	ns	ns	ns	ns	ns	ns

Supplementary Table 15 Statistical Test Applied in all figures

Figure	Measurement Type	Statistical Test Used
**Figure 1B**	IHC (EPX+ cells)	Mann–Whitney U
**Figure 1C–E**	IHC (EMT markers)	Mann–Whitney U
**Figure 1F**	Western blot (EMT proteins)	Kruskal-Walli's test; Dunn's multiple comparisons test
**Figure 1G–H**	ELISA (cytokines)	Kruskal-Walli's test; Dunn's multiple comparisons test
**Figure 2A–F**	IHC (fibrosis markers)	Mann–Whitney U
**Figure 2G**	Western blot (fibrosis markers)	Kruskal-Walli's test; Dunn's multiple comparisons test
**Figure 2H**	ELISA (TGF-β)	Kruskal-Walli's test; Dunn's multiple comparisons test
**Figure 3A–C**	IHC (mucus/goblet cell markers)	Mann–Whitney U
**Figure 3D–E**	Lung function (RI, Cdyn)	Kruskal-Walli's test; Dunn's multiple comparisons test
**Figure 3F**	ELISA (IL-13)	Kruskal-Walli's test; Dunn's multiple comparisons test
**Figure 4B–D**	IHC (fibrosis markers)	Mann–Whitney U
**Figure 4E**	Western blot (fibrosis markers)	Kruskal-Walli's test; Dunn's multiple comparisons test
**Figure 4F**	ELISA (IL-13)	Kruskal-Walli's test; Dunn's multiple comparisons test
**Figure 5B–E**	IHC (fibrosis markers)	Mann–Whitney U
**Figure 5F**	Western blot (fibrosis markers)	Kruskal-Walli's test; Dunn's multiple comparisons test
**Figure 6A–B**	IHC (goblet cells)	Kruskal-Walli's test; Dunn's multiple comparisons test
**Figure 6C–D**	Lung function (RI, Cdyn)	Kruskal-Walli's test; Dunn's multiple comparisons test
**Figure 6E**	ELISA (IL-13)	Kruskal-Walli's test; Dunn's multiple comparisons test
