# Supplementary material for: MMP-12 Inhibitors Inverse Eosinophilic Inflammation-Mediated Bronchial Fibrosis in Murine Models of Pulmonary Airway Obstruction
Source: Cells. 2025 Aug 23;14(17):1307. doi: 10.3390/cells14171307 (PMC12428497; doi:10.3390/cells14171307)
Supplement: Supplementary file 1 [file cells-14-01307-s001.zip › Supplementary figure legends.pdf]

Supplementary figure legends

Figure S1. MMP-12 expression correlates with eosinophilic inflammation in chronic allergen exposure. Representative lung tissue sections from saline-treated, allergen-challenged (Af), and MMP-12 knockout (MMP-12−/−) mice were examined to assess eosinophilic inflammation and epithelial–mesenchymal transition (EMT) markers. (i) Immunohistochemistry for eosinophil peroxidase (EPX) revealed strong eosinophilic infiltration in Af -exposed wild-type (WT) mice (i, b), which was markedly reduced in MMP-12−/− and Af-challenged MMP-12−/− groups (i, c - d) compared to saline controls (i, a). (ii) E-cadherin staining (epithelial marker) was decreased in Af -treated WT mice but partially preserved in MMP-12-deficient mice. (iii–iv) N-cadherin and vimentin (mesenchymal markers) were significantly increased in Af-challenged WT mice, while this effect was attenuated in MMP-12−/− lungs. All sections were visualized at 400× magnification. Quantification of EPX+ cells (i-e), E-cadherin+ cells (ii-e), N-cadherin+ cells (iii-e), and vimentin+ cells (iv-e) per mm^2 is shown. Data are presented as mean ± SEM (n = 4 mice/group) *$p < 0.05$, **$p < 0.01$, ***$p < 0.001$, **** $p < 0.0001$.

Figure S2. MMP-12 deficiency attenuates airway remodeling and fibrosis-associated marker expression in allergen-induced lungs. Representative lung sections from wild-type (WT) and MMP-12-deficient (MMP-12−/−) mice treated intranasally with saline or Aspergillus (Af) extract were stained and analyzed. (i) Immunohistochemistry (IHC) for MMP-12 shows strong expression in ASP-treated WT lungs (b) compared to saline (a), with significantly reduced expression in MMP-12−/− groups (c, d). (ii) IHC for FSP1, a fibroblast marker, demonstrates elevated expression in Af -treated WT lungs (b) and decreased staining in MMP-12−/− mice (d). (iii) Masson's trichrome staining highlights increased collagen deposition (blue) around airways in Af -exposed WT mice (b), which is markedly reduced in MMP-12−/− lungs (d). (iv) IHC for TGF-β reveals greater peribronchial staining in Af -exposed WT lungs (b), with reduced levels in knockout lungs (d). (v) IHC for α-SMA shows enhanced smooth muscle actin expression in Af -treated WT mice (b) indicative of myofibroblast activation, while knockout mice show reduced expression (d). (vi) IHC for SMAD4, a downstream mediator of TGF-β signaling, is elevated in Af-treated WT lungs (b) but significantly reduced in MMP-12−/− lungs (d). (e) Quantitative bar graphs corresponding to each staining represent mean ± SEM (n = 5–6 mice/group) of MMP-12+, FSP1+, collagen area (μm^2), TGF-β+, α-SMA+, and SMAD4+ cells per mm^2. Significant reductions in profibrotic markers were observed in MMP-12−/− mice compared to WT following Af-exposure. Data are presented as mean ± SEM (n = 4 mice/group). *$p < 0.05$, **$p < 0.01$, ***$p < 0.001$. All images are shown at 400× magnification.

Figure S3. MMP-12 deficiency attenuates allergen-induced goblet cell hyperplasia and mucin production in the lungs. Representative lung sections from wild-type (WT) and MMP-12-deficient (MMP-12−/−) mice treated with saline or allergen (Af) were analyzed to assess goblet cell hyperplasia and mucin expression. Periodic Acid–Schiff (PAS) staining (i) revealed a marked

increase in goblet cells in Af -treated WT mice compared to saline controls, which was significantly attenuated in MMP-12^−/− mice. Immunohistochemistry for MUCIN-1 (ii) and MUC5AC (iii) showed robust upregulation of these mucins in Af-challenged WT mice, whereas MMP-12−/− mice exhibited significantly lower expression levels. All images were captured at 400× magnification. Quantitative analysis of PAS+ goblet cells (i-e), MUCIN-1+ cells (ii-e), and MUC5AC+ cells (iii-e) per mm^2 demonstrated a significant reduction in mucin-producing cells in MMP-12-deficient mice following Af- exposure. Data are presented as mean ± SEM (n = 4mice/group). $^{**}p < 0.01$, $^{***}p < 0.001$, $^{****}p < 0.0001$.

Figure S4. MMP-12 deficiency mitigates IL-13–induced pulmonary fibrosis and myofibroblast activation in doxycycline-inducible transgenic mice. Lung sections from doxycycline (Dox)-inducible CC10-IL-13 and CC10-IL-13/MMP-$12^{-/-}$ bi-transgenic mice, with or without Dox administration, were analyzed for fibrosis and profibrotic markers. Masson's trichrome staining (A, i) shows extensive collagen deposition (blue) in Dox-treated CC10-IL-13 lungs (b) compared to untreated (a), while collagen staining is markedly reduced in Dox-treated CC10-IL-13/MMP-$12^{-/-}$ lungs (panel d) compared to their untreated counterparts (c). (A, ii) Immunohistochemistry (IHC) for α-SMA demonstrates increased myofibroblast activation in the Dox-induced CC10-IL-13 group (b), which is significantly attenuated in the absence of MMP-12 (d). (A, iii) IHC for TGF-β reveals upregulation in Dox-induced CC10-IL-13 lungs (b), while the Dox-treated CC10-IL-13/MMP-$12^{-/-}$ group exhibits reduced expression (d), indicating a dampened fibrotic response. Quantitative graphs (e) show significant increases in collagen area (μm^2), α-SMA^+, and TGF-β^+ cell counts in Dox-treated CC10-IL-13 lungs, with substantial reductions in the CC10-IL-13/MMP-$12^{-/-}$ mice upon Dox exposure. Data are presented as mean ± SEM (n = 4 mice/group), $^{****}p < 0.0001$. All images captured at 400× magnification.

Figure S5. Pharmacological inhibition of MMP-12 attenuates allergen-induced lung fibrosis and expression of profibrotic markers. Lung sections from mice treated with saline, Aspergillus (ASP) extract, MMP-12 inhibitors (MMP408 or PF-HCL), or a combination of ASP with MMP-12 inhibitors were analyzed to assess the effect of MMP-12 inhibition on allergen-induced pulmonary fibrosis and profibrotic responses. Masson's trichrome staining (i) shows marked collagen accumulation in ASP-treated lungs (b), which is significantly reduced following treatment with either MMP408 (d) or PF-HCL (f). Immunohistochemistry for α-SMA (ii) reveals increased myofibroblast activation in ASP-treated mice, with reduced α-SMA expression in the inhibitor-treated groups. MMP-12 staining (iii) confirms strong induction by ASP, which is suppressed by MMP408 and PF-HCL. Similarly, TGF-β staining (iv) is elevated in ASP-treated lungs and reduced upon MMP-12 inhibitor treatment. Quantitative analysis (i-iv, g) demonstrates that collagen area, and numbers of α-SMA^+, MMP-12^+, and TGF-β^+ cells are significantly increased in ASP-exposed lungs and significantly attenuated with either MMP408 or PF-HCL treatment. Eosinophils were also showed by EPX attaining (v), followed by quantification. Data represents mean ± SEM (n = 4 mice/group), $^{****}p < 0.0001$. All images were acquired at 400× magnification.

Figure S6. The immunofluorescence analysis for anti-MMP-12, anti-F4/80 on the lung tissue sections of saline and Af-challenged mice. DAPI mounted merged photomicrograph of saline (A-D) and *Aspergillus* (E-H) are shown. Images were captured and presented in 400x of original magnification.

Figures S7–S10. All the original western blots.

Table S1. List of antibodies, their dilutions used in western blotting and immunohistochemistry.

Tables S2–S15. Statistical analysis that was carried out in each figure.
